# pH-Responsive Polymer Nanomaterials for Tumor Therapy

**DOI:** 10.3389/fonc.2022.855019

**Published:** 2022-03-22

**Authors:** Shunli Chu, Xiaolu Shi, Ye Tian, Fengxiang Gao

**Affiliations:** ^1^ Department of Implantology, Hospital of Stomatology, Jilin University, Changchun, China; ^2^ Changchun Institute of Applied Chemistry, Chinese Academy of Sciences, Changchun, China

**Keywords:** tumor, pH-responsive polymer, nanomaterials, tumor microenvironment, drug delivery

## Abstract

The complexity of the tumor microenvironment presents significant challenges to cancer therapy, while providing opportunities for targeted drug delivery. Using characteristic signals of the tumor microenvironment, various stimuli-responsive drug delivery systems can be constructed for targeted drug delivery to tumor sites. Among these, the pH is frequently utilized, owing to the pH of the tumor microenvironment being lower than that of blood and healthy tissues. pH-responsive polymer carriers can improve the efficiency of drug delivery *in vivo*, allow targeted drug delivery, and reduce adverse drug reactions, enabling multifunctional and personalized treatment. pH-responsive polymers have gained increasing interest due to their advantageous properties and potential for applicability in tumor therapy. In this review, recent advances in, and common applications of, pH-responsive polymer nanomaterials for drug delivery in cancer therapy are summarized, with a focus on the different types of pH-responsive polymers. Moreover, the challenges and future applications in this field are prospected.

## 1 Introduction

Cancer is a major cause of morbidity and mortality, greatly impacts quality of life, and is a serious threat to human health; the number of diagnosed patients has increased gradually in recent years (1–4). For example, recently, there were 18 million new cancer cases and 9.5 million cancer deaths worldwide in 2018 (1). Many scientists worldwide are committed to researching the diagnosis and treatment of cancerous tumors (5–10). Although this research has achieved some results, to some extent, the complex tumor microenvironment remains a barrier to novel tumor therapy programs. Chemotherapeutic therapy, referred to as chemotherapy, is still one of the main methods of tumor treatment (11–13). Some chemical drugs, such as oxaliplatin, doxorubicin (DOX), and paclitaxel (PTX), are applied to kill tumor cells and shrink or destroy the tumor (14–18). Because drug delivery is generally given systemically, the body itself will also be affected and damaged to some extent, causing side effects, complications, and even death in some serious cases (19–22). Therefore, the development of target-specific or stimuli-sensitive drugs that can identify tumor cells, to achieve the purpose of tumor treatment with no or very little impact on normal tissue cells, is the current focus of research.

During tumor growth, cell proliferation and angiogenesis are uncontrolled and dysfunctional, resulting in a complex tumor microenvironment, comprising severe hypoxia, a slightly acidic pH, and excessive ROS (22–24). Owning to the severely hypoxic microenvironment, a large number of acid metabolites are produced through glycolysis in tumor cells, leading to a weakly acidic microenvironment (pH < 6.5) around tumor tissues, which is lower than the pH value of blood and healthy tissues (pH 7.4) (25–27). In addition, after endocytosis, the rapid acidification due to proton influx decreases the pH values of intracellular and suborganelle structures (pH 5.0–6.0) and lysosomes (pH 4.0–5.0) (28, 29).

This complex tumor microenvironment can be exploited by researchers to obtain specific responses; for example, pH-responsive stimuli were found to respond to the difference in pH between normal and tumor tissues (30, 31). pH-responsive stimulation is a powerful tool that can be used to regulate the properties of materials (32). Researchers have designed pH-responsive polymer nanomaterials (10–100 nm) (33) that can be engineered to suit the need of the therapy and target area. These materials are relatively stable in the blood and normal tissue but are unstable in the tumor area (acidic pH), leading to rapid changes in the degree of ionization in pH-responsive groups, causing material changes that release the anti-tumor drugs, achieving the goal of targeted tumor therapy (34, 35). Researchers have performed sustainable research on these pH-responsive polymer nanomaterials (36, 37). This review will focus on pH-responsive polymer nanomaterials in tumor therapy and comprises a brief introduction to anti-tumor drug loading methods, a clarification of the mechanism and application of pH-responsive polymer nanomaterials in tumor therapy, an outlook addressing the great recent challenges faced, and suggestions for applications and future research directions.

## 2 Anti-Tumor Drug Loading Methods for pH-Responsive Polymer Nanomaterials

**Figure 1:**
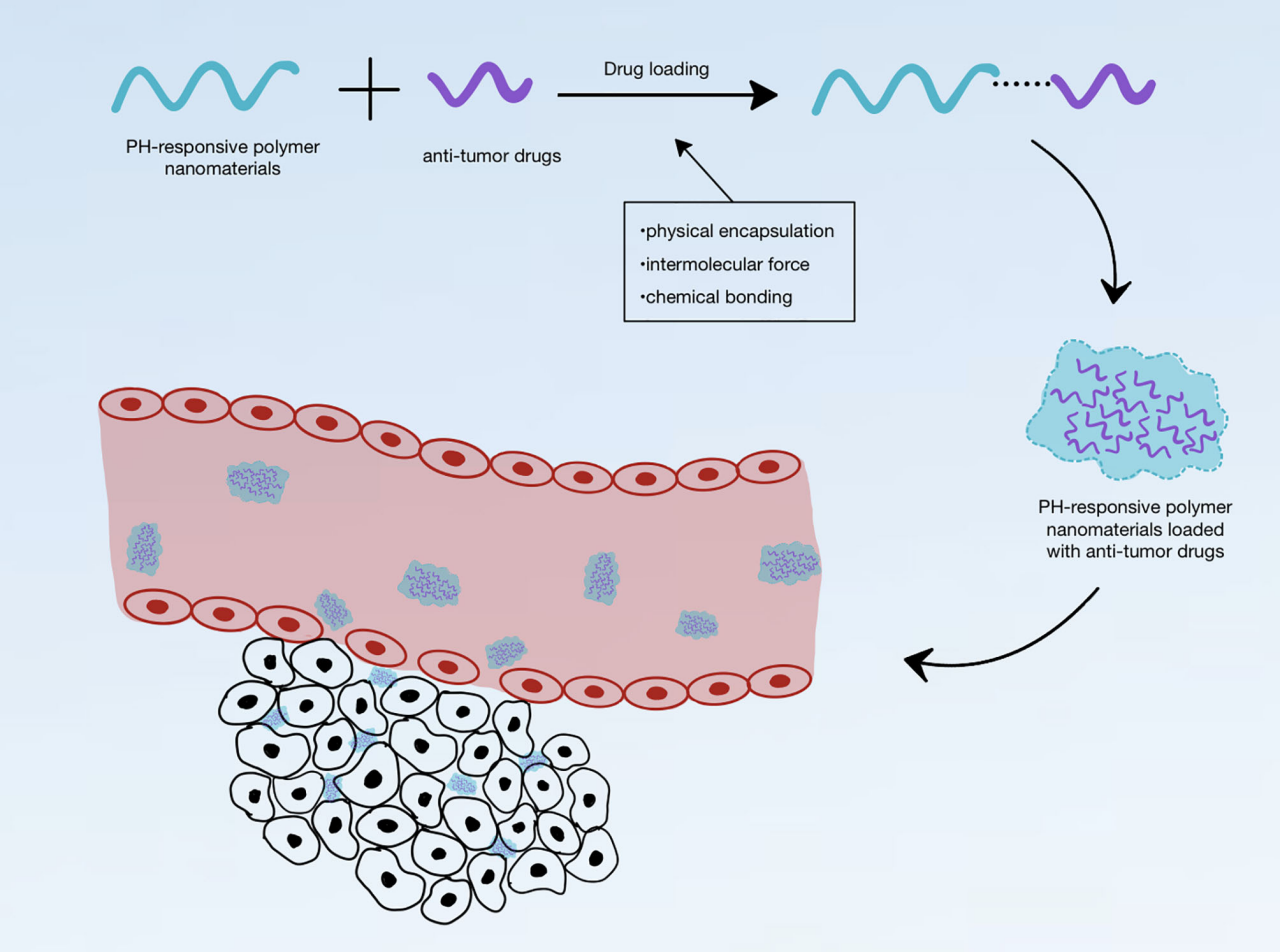
Schematic diagram of pH-responsive polymer nanomaterials loaded with anti-tumor drugs and entering the tumor environment.

Theoretically, pH-responsive polymer nanomaterials can carry anti-tumor drugs, which are transported into the tumor tissue and are then released to produce therapeutic effects on tumors. pH-responsive nanomaterials are classified into organic nanomaterials (liposomes, polymer micelles, polymer capsules, nano-gels, dendrimers, etc.), inorganic nanomaterials [carbon nanoparticles (NPs), silica NPs, gold NPs, etc.], and composite nanomaterials [metal–organic frameworks (MOFs) and metal-phenolic networks, etc.] (**Table 1**). They exhibit many different structures: linear (homopolymer, biblock copolymers, multiblock copolymers, and organic/inorganic hybrid polymers), non-linear branched (branched or hyperbranched polymers), and grid. Linear homopolymers and amphiphilic and amphiphilic block copolymers the most common types, and mainly form micelles, vesicles, dendritic macromolecules, and nanogels. Owing to the different properties of different drug molecules, it is necessary to select the appropriate nanomaterial according to the properties of the particular drug; drugs and nanomaterials can be combined in different ways. The anti-tumor drug loading methods for pH-responsive polymer nanomaterials can be classified into three categories: physical encapsulation, intermolecular force, and chemical bonding, *via* the connection between the pH-responsive polymer nanomaterial and anti-tumor drug (**Figure 1**).

**Table 1:** Classification of nanomaterials.

Nanomaterials				
Classify			Morphology or structure	Characteristics or deficiency
Organic Nanomaterials	Liposomes		A hydrophobic phospholipid bilayer, and a central hydrophilic cavity	Low toxicity, good biocompatibility, easy degradation; poor stability *in vitro*, and *in vivo*, and unstable drug release kinetics
	Polymer capsules		A hydrophobic wall, and a hydrophilic cavity	
	Polymer micelles		“Core-shell” structure, hydrophobic cores, and hydrophilic shell	
	Nanogels		Colloidal particles, with an internal cross-linked structure	
	Dendrimers		Spherical structures of multi-branched polymers, central cores, branches of repetitive units, and an outer layer of multivalent functional groups	
	etc.			
Inorganic Nanomaterials	Carbon nanoparticles	Graphene	Network structure from zero-dimensional to three-dimensional, and at least one dimension of the dispersion size is less than 100 nm	Good biocompatibility, electrical conductivity, optical properties, and photothermal properties; carrier surface modification is very important
		Carbon nanotubes		
		Fullerenes		
		Carbon quantum dots		
		etc.		
	Silica nanoparticles		Mesoporous silica nanoparticles, a large number of pore structures, the aperture is in the range of 2-50 nm	The ordered arrangement of pores, tunable pore size, pore volume, and surface chemistry, high chemical stability, and biocompatibility; rely on surface modification to achieve the controlled release of drug molecules
	Metallic nanoparticles	Gold nanoparticles	Core (metal), and shell (functional material)	Gold nanoparticles have a wide range of applications, controllable particle size, easy surface modification, good biocompatibility, excellent photothermal properties, and enzyme activity
		Silver nanoparticles		
		Iron nanoparticles		
		etc.		
	etc.			
Composite Nanomaterials	Metal-Organic Frameworks		An infinitely extended network-like structure, organic bridging ligands linked to metal ions (clusters) by self-assembly or coordination bonds	Large surface area, adjustable performance, a large amount of drug encapsulation, and sustained release effect
	Metal-Phenolic Networks		A network structure formed by the coordination of phenolic hydroxyl groups with various metal ions in polyphenols	Good biocompatibility; poor biological antifouling performance
	etc.			

### 2.1 Physical Encapsulation

Physical encapsulation is the earliest and most effective drug loading method for nanomaterials. It can be used in organic, inorganic, and composite nanomaterials; the most common nanomaterials discussed for its use in the literature are liposomes, polymer micelles, polymer capsules, mesoporous silica NPs, MOFs, and metal–polyphenol nano-networks.

Liposomes were the earliest kinds of nano-drug. They mainly consisted of a bilayer of phospholipid molecules, which could be used to encapsulate the anti-tumor drugs in a hydrophobic phospholipid bilayer or a central hydrophilic cavity; their drug piggybacking was achieved through the physical encapsulation route. Hong et al. (38) designed lipid–polymer hybrid NPs with poly(lactic-co-glycolic acid) as the hydrophobic core to encapsulate afatinib and/or miR-139 encapsulated in an amphiphilic shell of polyethylene glycol (PEG) liposomes, and further modified the shell layer with ligand arginine and pH-sensitive cell-penetrating peptide histidine to enhance the targeting and penetration of these multifunctional NPs in the acidic microenvironment of colorectal cancer cells. Similar to iposomes, physical encapsulation is the main method of drug loading polymer capsules; the polymeric capsules encapsulate hydrophobic drug molecules within the capsule wall and hydrophilic drug molecules in the cavity (39). Zhong et al. (40) synthesized a pH-responsive block polymer, which comprised methoxy PEG (mPEG) and poly(asparagyl diisopropylethylenediamine-co-phenylalanine), self-assembled into nano-microcapsules as a drug carrier, and encapsulated the hydrophilic hypoxia-activated prodrug tirapamine and hydrophobic photosensitizer dihydroporphyrin [chlorin e6 (Ce6)] to treat breast cancer effectively. Polymeric micelles are the most common carriers of high-molecular-weight polymers. They are thermodynamically stable colloidal solutions formed by the self-assembly of amphiphilic block copolymers in water, and their “core-shell” structure can encapsulate hydrophobic drugs in hydrophobic cores. Xu et al. (41) encapsulated the reactive oxygen species (ROS) generator vitamin K3 with pH/ROS dual-sensitive polymer prodrug PEG-b-P[(LL-g-TK-PTX)-(LL-g-DMA)] micellar NPs to prepare polymeric prodrug-based drug delivery systems (DDSs) with pH-induced surface charge inversion and ROS amplification for ROS-triggered self-accelerated drug release. Mesoporous silica NPs (MSNs) contain a large number of pore structures, which can significantly increase the drug loading capacity and can be blocked by blocking agents, such as surface films or pH-sensitive ZnO quantum dots (QDs), to prevent the drug from leaking at non-targeted positions during transportation *in vivo*. The inorganic material on the walls of MSNs can prevent mechanical, thermal, and biological degradation of the encapsulated drugs to provide safe and effective drug delivery (42). Yan et al. (43) developed a biodegradable pH-responsive hollow MSN for the co-delivery of pheophorbide a and DOX. The NP possessed controllable particle size and a large inner hollow core, giving it excellent encapsulation capacities. MOFs are crystalline materials consisting of organic bridging ligands linked to metal ions (clusters) employing coordination bonds to form an infinitely extended network-like structure, which can physically embed drugs. He et al. (44) loaded cisplatin precursor drugs into UiO nanoscale MOFs with hexagonal-plate morphologies by encapsulation and loaded multiple drug resistance gene-silencing siRNAs by the surface ligand to enhance the therapeutic efficacy against drug-resistant ovarian cancer cells. Liposomes and nanocapsules can carry drugs only by physical encapsulation, unlike other carriers, which can carry drugs through physical encapsulation, using intermolecular forces, or *via* chemical bonding.

### 2.2 Intermolecular Forces

The use of intermolecular forces is also a common non-chemical bonding method of attaching drugs to carriers. Such forces include electrostatic attraction, hydrogen bonding, π–π interaction, van der Waals forces, and hydrophobic interactions. For water-soluble polymers, drugs can be loaded onto nanomaterials by intermolecular interaction to increase their circulation time and limit their toxicity to normal tissues (45–48). Polymer micelles, nanogels, carbon NPs, and dendritic macromolecules can be combined with drugs *via* one or more intermolecular forces. Polymeric micelles are capable of carrying drug molecules in their cores using hydrophobic forces and hydrogen bonding forces. Wu et al. (49) wrapped hydrophobic DOX in the core of core/shell PEGylated poly(glycerol sebacate) (PEGS)/hydroxyapatite hybrid nanomicelles using hydrogen bonding and fabricated spherical NPs with a high drug loading capacity and pH-sensitive response release by optimizing the coordination of PEG nanomicelles with the hydroxyapatite mineralization-responsive release of spherical NPs. In addition, multi-intermolecular forces can stabilize the bond between carriers and drugs. Zhao et al. fabricated co-loaded NPs using supramolecular assembly, which consisted of a block copolymer of mPEG and polycarbonates, DOX, and Ce6. DOX and Ce6 were mainly dispersed in the hydrophobic core. The hydrophobic polycarbonate chain segments and cationic polycarbonate chain segments generated strong intermolecular interactions between the polymer and cargo, including hydrophobic interactions, hydrogen bonding interactions, and electrostatic interactions. The strong interactions between Ce6 and DOX, including electrostatic interactions, π–π stacking interactions, and hydrophobic interactions, favored the formation of NPs (50). Nanogels with an internal cross-linked structure often adsorb drug molecules through hydrophobic interactions and can use their swelling and anti-swelling characteristics to achieve the stimulus-responsive release of drug molecules. Howaili et al. (51) synthesized a plasmonic nanogel loaded with curcumin (AuNP@Ng/Cur) as a stimuli-responsive nanocarrier, using an incubation method at temperatures above the lower critical solution temperature to promote hydrophobic interactions between the Cur and nanogel to achieve loading. Dendrimers are well-defined spherical structures of multi-branched polymers, characterized by central cores, branches of repetitive units, and an outer layer of multivalent functional groups. Their hydrophobic core can encapsulate uncharged non-polar small molecular drugs, and their functional groups can electrostatically interact with charged polar molecules or chemically bind with immunotherapeutic agents such as therapeutic antibodies. Shi et al. (52) developed a unique polymeric hybrid NP nanoplatform for multi-stage siRNA delivery, which was formed by electrostatic interactions between the copolymer mPEG–poly(l-histidine)-poly(sulfadimethoxine), the dendrimer poly-l-lysine, and PLK1siRNA to efficiently deliver siRNA.

### 2.3 Chemical Bonding

Researchers often combine drugs with carriers through chemical bonds, such as coordination bonds and acid-unstable bonds, and common nanomaterials, including composite nanomaterials (MOFs), organic nanomaterials (dendrimers), and inorganic nanomaterials (quantum dot nanomaterials).

MOFs are composite nanomaterials with intramolecular pores formed by the self-assembly of organic ligands and metal ions or clusters through coordination bonds (53). Shen et al. (54) prepared a core-shell nanocomposite composed of zeolite imidazole skeleton-90 (ZIF-90) coated with spermine-modified acetal dextran (SAD), in which hydrophilic DOX was covalently bonded to the ZIF-90 skeleton and the hydrophobic photosensitizer IR780 was loaded into the SAD shell, thus allowing combined photodynamic therapy and chemotherapy. Duan et al. (55) fabricated a novel pH-responsive MOF antigen-delivery system to solve the problems in the delivery strategy for tumor-associated antigens (TAAs). Through the one-pot synthesis process, TAAs were loaded into MOFs, which were released into the tumor site and disintegrated due to hydrolysis caused by the slightly acidic tumor environment, thus enhancing the anti-tumor effect. In dendrimer polymers, such as PAMAM, functional groups can be chemically linked with immunotherapeutic agents. Xia et al. (56) linked DOX to the terminal amino group of PAMAM through a pH-sensitive hydrazone bond [PEG-PAMAM-DOX (PPD) conjugate] and designed a PAMAM-based NP (LMWH/PPD/CpG) loaded with DOX and immunoadjuvant cytosine–phosphate–guanine oligonucleotides (CpGODNs) for the combined chemotherapy and immunotherapy of metastatic melanoma. QDs in inorganic nanocarriers can also combine drugs with carriers through special coordination. Cai et al. (57) synthesized the first acid-decomposable, luminescent aminated ZnO QDs as nanocarriers with ultra-small size, modified with dicarboxyl-terminated PEG and hyaluronic acid (HA), and finally, constructed a ZnO QD-based pH-responsive drug delivery platform, in which DOX molecules were successfully loaded to PEG-functionalized ZnO QDs *via* the formation of a metal–DOX complex and covalent interactions. After the ZnO QDs are taken up by cancer cells, the metal–drug complexes dissociate in the acidic environment of the endosomes or lysosomes, releasing Zn2+ and DOX, achieving synergistic therapy.

In terms of drug loading rate and accurate controlled drug release, the performance of physical encapsulation is poorer than that of the other two drug-loading methods, which can not only reduce the non-specific release of drugs during circulation, but can also increase drug loading by adjusting the ratio of reactants and have obvious advantages in clinical production and application. These benefits may be related to the molecular weight, surface and internal structures of the material, whether the surface is charged, and whether the surface is hydrophobic. Owing to the advantages and disadvantages of these three different loading methods and the complexity of the tumor microenvironment, researchers have constructed nanomaterials with a diverse combination of different drug-loading methods and achieved satisfactory results in drug delivery and tumor treatment. Two pH-sensitive conjugates, pluronic P123-mono–hydrazone bond-P123-single-hydrazone–docetaxel and pluronic P123-double–hydrazone bond–docetaxel, prepared by Su et al. (58) were used as carrier materials to encapsulate docetaxel physically. These polymer micelles, which were prepared using chemical combination and physical encapsulation, can increase the drug loading capacity and drug release control compared to those created by chemical combination or physical encapsulation.

## 3 Mechanism of pH-Responsive Polymer Nanomaterials in Tumor Therapy

pH-responsive polymer nanomaterials carrying anti-tumor drugs can change their structures or properties with the pH reduction in the tumor microenvironment, thus providing accurate targeted tumor therapy. The common pH-sensitive structures mainly include chemical bonds, which are relatively stable in a neutral or alkaline environment but unstable in an acidic environment and are hydrolyzed or broken; polymers that can change their charge properties according to the change in pH (59); and other special pH-responsive polymers. Since these special pH-responsive structures will change their structures or properties according to changes in pH in the environment, the structure of nano-drug carriers containing these structures will also change (rearrangement, expansion, or degradation) (60). The aim of this is to release the loaded therapeutic drugs, remove the protective layer, cause disintegration close to targeted molecules in areas of pH change, and overall, achieve improved drug delivery efficiency and therapeutic effects (61; 62).

### 3.1 pH-Sensitive Chemical Bonds

**Figure 2:**
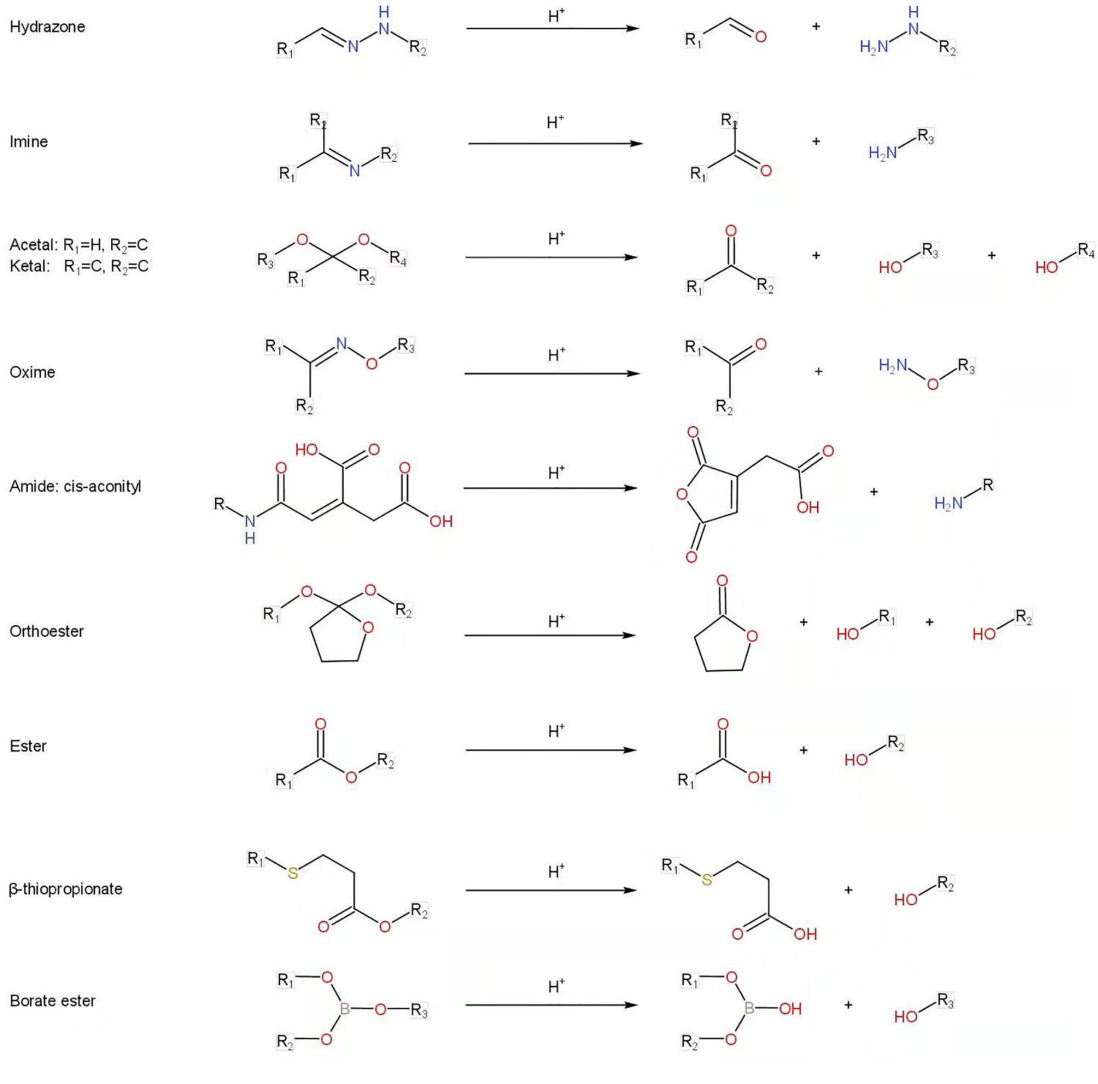
PH-responsive linkages and the corresponding hydrolyzed products.

Previously, we have discussed the drug loading methods for nanomaterials. Although the special spatial structure of some nanomaterials allows for the physical loading of drug molecules, combining the drugs and polymer carrier through chemical bonds can better control the distribution and release of drugs, can make the nano-drug system more stable *in vivo*, and only specifically degrade and release drugs in the tumor tissue environment (63–66). At present, we already know that many chemical bonds are unstable and easy to break in a strongly acidic or strongly alkaline environment. We can utilize these unstable chemical bonds in an acidic environment and introduce them into nano-drug carriers to construct a pH-responsive polymer DDS that can specifically release drugs in the slightly acidic tumor microenvironment. The common pH-sensitive bonds mainly include hydrazone/acyl hydrazone, imine, acetal/ketal, oxime, amide, ester/orthoester, β-thiopropionatent, and borate ester (59, 60, 67–69). The general structures of these bonds and the corresponding hydrolyzed products are summarized in **Figure 2**.

#### 3.1.1 Hydrazone/Acyl Hydrazone Bonds

Hydrazone/acyl hydrazone bonds are formed by the condensation reaction of hydrazine groups (-NH-NH2) or hydrazide groups (-CONH-NH2) with ketones or aldehydes. This reaction is relatively stable at pH 7.4, and thus, the hydrolysis rate is very slow but will be accelerated at lower pH levels (70). Based on this, hydrazone/acyl hydrazone bonds are widely used in drug delivery (71).

In the early 1990s, Greenfield et al. (72) and Kaneko et al. (73) had studied various hydrazone bonds formed by the condensation of the ketone groups of DOX with hydrazide groups. The sensitivity of the hydrazone bond to the acidic environment has since been deeply and extensively studied, and it has been applied to various structures, including linear (73), star-shaped (74), and cross-linked (75) molecules. Recently, Long et al. (76) reported a novel strategy to endow cellulose nanocrystals (CNCs) with pH responsiveness and drug loading through the formation of hydrazone bonds between PEG-modified CNCs and the aldehyde group of DOX. DOX was loaded on PEG-modified CNCs and could be specifically released from pH-responsive drug polymer carriers at the tumor site. The results of anti-tumor experiments both *in vivo* and *in vitro* showed that the drug carrier had obvious cytotoxicity and a good anticancer effect.

The most common method of introducing hydrazone bonds into nano-drug carriers is the condensation of hydrazide groups in various polymers with ketones or aldehyde group drugs. Therefore, the existence of ketone or aldehyde functional groups in drug molecules is the main challenge hindering the application of hydrazone bonds (77). Although many researchers have synthesized drug derivatives to obtain ketones or aldehyde groups, such as PTX synthesized by Alani et al. (78), cisplatin synthesized by Aryal et al. (79), and Cur derivatives synthesized by Li et al. (80) and Wang et al. (81) in clinical application, the product development and regulatory approval of these drug derivatives are difficult in practice (77). At the same time, the cationic polymer residues that may be formed after the hydrazone bond breaks in an acidic environment will produce cytotoxicity to a certain extent (82). These limitations in the application of nano-polymers containing hydrazone bonds to tumor therapy require improvement through further research.

#### 3.1.2 Imine Bonds

The structural formula of imine bonds is -C=N-, which is widely known as a pH-sensitive dynamic covalent bond. Its carbon–nitrogen double bond structure is obtained by the condensation reaction of aldehydes or ketones with primary amines, i.e., the **“**Schiff base reaction**”**. Therefore, the Schiff base structure mainly refers to a class of compounds containing C=N groups. Although it has been proven that the structure can exist stably in an alkaline or neutral environment and accelerate the hydrolysis/breaking rate in an acidic environment (83, 84), some researchers agree that imide bonds are not stable enough under physiological conditions (i.e., pH 7.4 and 37°C) (85, 86). Thus, it has been suggested that increasing the stability by conjugating with the π–π bond to form benzoic–imide bonds could be a solution to this (87).

The synthesis of benzoic–imide bonds involves amines and benzaldehydes, which exist naturally in many raw materials and are easy to obtain and synthesize. They are very suitable for constructing pH-responsive polymer nanomaterials and have been widely used in anti-tumor DDSs. For example, Liao et al. (88) prepared pH-responsive benzoic-imide polymer nanogels as carriers of indocyanine green (ICG) by crosslinking poly(ethylenimine)-g-mPEG (PEI-g-mPEG) copolymers with hydrophobic terephthalaldehyde molecules in an aqueous solution with pH 7.4. The nanogel loaded with ICG not only significantly improved the photostability of ICG in phosphate buffer, but also clearly slowed down the leakage of ICG at pH 7.8, and when the pH was decreased from 7.8 to 6.4, the benzoic–imide bonds in the nanogel broke and released ICG, showing targeted release.

#### 3.1.3 Acetal/Ketal Bonds

The structural formula of acetals/ketals allows for two alkoxy groups to connect on the same carbon; the difference between them is that ketals have one more central carbon substituent than acetals. Under acidic conditions, one of the oxygen atoms is protonated, thus activating the adjacent carbon atoms and leading to the hydrolytic fracture of bonds. Previous studies have shown that polymers containing acetals/ketals can be degraded into soluble monomers in the acidic pH of a tumor microenvironment (89, 90).

Acetal/ketal bonds are also widely used in pH-responsive polymer nano-DDSs (NDDSs). Zhao et al. (91) polymerized cinnamaldehyde (CA) directly with glucose through acid-unstable acetal bonds and encapsulated 10-hydroxy camptothecin (HCPT), and then, synthesized a novel type of pH-responsive DDS comprising HCPT–CA-loaded NPs (PCH). In an acidic environment, both HCPT and CA could be rapidly released by PCH. The results of anti-tumor experiments both *in vivo* and *in vitro* showed that PCH could not only effectively prolong the drug circulation, increasing the drug accumulation in the tumor site and promoting the intake of drugs, but also induced the death of cancer cells by producing intracellular ROS, showing excellent therapeutic performance and improved biosafety.

#### 3.1.4 Oxime Bonds

The structural formula of oxime bonds is -C=N-O-. These bonds are reversible dynamic covalent chemical bonds, similar to imine bonds. The common method for preparing oxime bonds is a condensation of aldehydes or ketones with hydroxylamine groups, which can be hydrolyzed into aldehydes, ketones, and hydroxylamines under acidic conditions (92, 93). Studies have shown that oxime bonds are reversible, have dynamic pH sensitivity, and can form a new type of pH-responsive polymer (94). Jin et al. (94) successfully synthesized a pH-responsive triblock copolymer (PEG-OPCL-PEG) by ligating aminooxy terminals of OPCL with aldehyde-terminated PEG (PEG-CHO), which consisted of hydrophilic PEG and hydrophobic oxime-tethered polycaprolactone (OPCL). DOX was selected as an anti-tumor model drug, which was encapsulated in PEG-OPCL-PEG and self-assembled into micelles in an aqueous solution. The drug release studies showed that the release rate of DOX in micelles at pH 5.0 was significantly faster than that at pH 7.4, which indicated that the oxime bond DDS had the characteristic of pH response. The results of anti-tumor experiments both *in vitro* and *in vivo* also showed that DOX-loaded PEG-OPCL-PEG micelles had a high anti-cancer effect.

#### 3.1.5 Amide Bonds

Amides are an important part of pharmaceutical chemistry, and more than 25% of drugs in the database of pharmaceutical chemistry contain amide bonds (95). The structural formula of amide bonds is (-C=O-N-). The synthesis method involves initial preparation using active carboxylic acid derivatives, such as acyl chloride, anhydride, ester, and azide, followed by development into an acylated preparation using substrates such as alcohols, aldehydes, and alkynes, catalyzed by new catalysts and amines (96, 97). Because some amide bonds with special side chains are hydrolyzed and broken under weakly acidic conditions (98, 99), such as maleic acid (MA) (60), cis-aconitine acid (100), and dimethyl maleic anhydride (DMMA) (101), amide bonds are also widely used in pH-responsive polymer NDDSs. Luo et al. (102) linked stearic acid (SA) to the main chain of carboxymethyl chitosan (CMC) through amide bonds, which then self-assembled into an amphiphilic stearic acid-O-carboxymethyl chitosan conjugate (SA-CMC), and then loaded the anti-tumor drug PTX onto the conjugate to form PTX-SA-CMC NPs. In the acidic environment of the tumor, the NPs disintegrated rapidly and released drugs specifically, owing to the pH sensitivity or acid instability of amide bonds. Both *in vivo* and *in vitro* experimental results showed that compared to those in the free PTX group, the drug uptake rate of tumor cells and killing effect in the PTX-SA-CMC NP group were significantly enhanced, which not only reduced the systemic toxicity but also achieved a noticeable anti-tumor effect.

#### 3.1.6 Ester Bonds

The structure of the ester bonds or orthoester bonds is similar, in which one carbon atom is connected with two or three oxygen atoms. They are also pH-sensitive and degrade faster than acetals, ketals, or hydrazone bonds in an acidic environment (103, 104). At present, ester bonds or orthoester bonds have been widely studied and applied in pH-responsive polymers. Thambi et al. (105) prepared pH-sensitive amphiphilic block copolymers with orthoester bonds using hydrophilic PEG and hydrophobic poly(γ-benzyl-L-glutamate) (PBLG) as carriers and loaded DOX into them. *In vitro* release studies showed that DOX was released slowly from the carrier in the physiological buffer with pH 7.4, whereas the release rate of DOX increased significantly at pH 5.0. *In vitro* cytotoxicity tests showed that the toxicity of these DOX-loaded nanocarriers to SCC7 cancer cells was higher than that of DOX carriers without orthoester bonds. These results indicated that this pH-responsive copolymer NDDS with orthoester bonds has great potential in tumor therapy.

#### 3.1.7 β-Thiopropionate Bonds

Owing to the inductive effect of the S atom in the β-thiopropionate bond, the hydrolysis of the bond is driven by the positive charge on the carbonyl carbon of the ester. Compared with other pH-resbeponsive chemical bonds, such as hydrazone, imide, and acetal/ketal bonds, which are very sensitive to pH changes, and thus, have a high hydrolysis rate in an acidic environment, β-thiopropionate bonds can be hydrolyzed at a relatively slow rate in a moderately acidic environment (for example, pH 5.5) (106–108).

β-thiopropionate bonds can be synthesized by simple Michael addition reaction of mercaptan methacrylate (106, 107). Samanta et al. (69) copolymerized 2-hydroxyethyl methacrylate and a coumarin-based methacrylate monomer containing a β-thiopropionate moiety *via* reversible addition–fragmentation chain transfer (RAFT) polymerization. The copolymer self-assembled to form vesicular nanoaggregates at physiological pH conditions, and then, photochemical crosslinking was performed *via* coumarin (2π + 2π) cycloaddition reaction to further stabilize the vesicles. In the acidic environment of tumor cells, the slow hydrolysis of β-thiopropionic acid led to the disintegration of the polymers, resulting in the sustainable release of the loaded drugs. The results of the *in vivo* and *in vitro* anti-tumor experiments showed that compared with free DOX, the 50% inhibiting concentration (IC50) of DOX-loaded nanomaterials toward the MG63 cancer cell line decreased significantly, showing excellent anti-tumor effect and good biosafety.

#### 3.1.8 Borate Ester Bonds

Borate ester bonds, formed by the condensation of phenylboronic acid and its derivatives with cis-diol or compounds containing catechol groups, are also reversible dynamic covalent bonds. At acidic pH levels, borate ester bonds can be reversibly hydrolyzed and broken, which gives them broad application prospects in tumor DDSs (109–111). Fang et al. (112) prepared a mitochondrial-targeted polymer, 3,4-dihydroxyphenyl propionic acid-chitosan oligosaccharide-dithiodipropionic acid-berberine (DHPA-CDB), which was used to load Cur and self-assemble into cationic nano-micelles (DHPA-CDB/CUR). Negatively charged oligomeric hyaluronic acid-3-carboxyphenylboric acid (OHA-PBA) was added to the prepared DHPA-CDB/CUR as the ligand of sialic acid and CD44, to shield the positive charge and prolong the circulation time *in vivo*. The OHA-PBA@DHPA-CDB/CUR covalent polymer was formed by OHA-PBA and DHPA-CDB/CUR through pH-sensitive borate ester bonds between PBA and DHPA. The low pH in tumor tissue promoted the degradation of borate ester bonds and the exposure of cationic micelles, which led to the reversal of the polymer surface charge, thus promoting internalization and mitochondrial localization. All experimental results showed that OHA-PBA@DHPA-CDB/CUR had mitochondrial targeting and tumor environmental charge-reversal capabilities, as well as great potential in targeted drug delivery.

### 3.2 pH-Responsive Charge Conversion

It is well known that nano-drug carriers reach the target position mainly through blood circulation in the human body. If there is a positive charge on the surface of the drug carrier when it encounters the many negatively charged proteins in human plasma, they will attract each other strongly; this makes it very easy for the carriers to be swallowed and cleared by phagocytes in the human body (113, 114), resulting in reduced drug delivery efficiency. The drug uptake efficiency will be reduced due to electrostatic repulsion when a drug carrier with a neutral or electronegative surface encounters the electronegative cell membrane (115, 116), whereas electropositive nano-drug carriers can better target endothelial cells, have higher vascular permeability (117), and are more likely to be swallowed by tumor cells (118–120). However, the liver can act as a charge-selective filtration barrier, which can cause the accumulation of electronegative nano-drug carriers in the liver with consequential potential side-effects. Thus, the long-cycle ability of neutral polymers or amphoteric ionic polymers is a better option (121).

**Figure 3:**
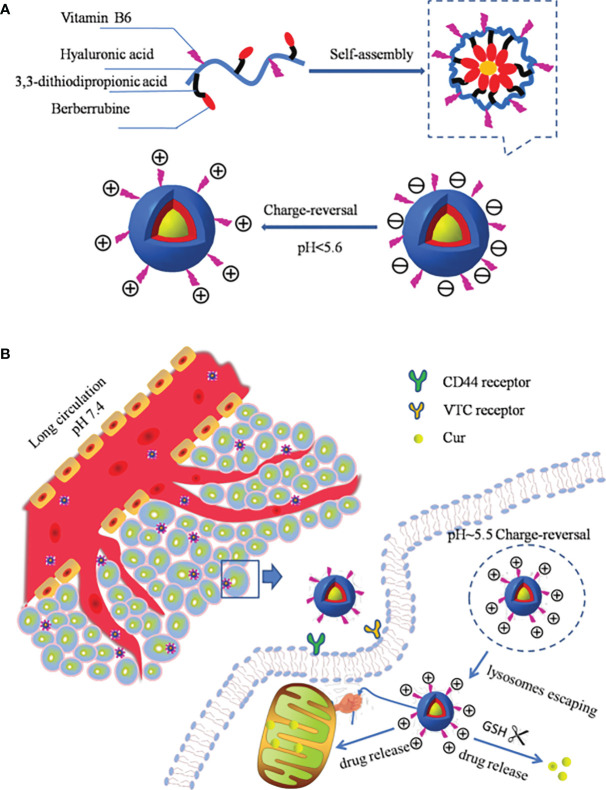
**(A)** Schematic structure of B6-OHA-SS-Ber self-assembly into micelles; **(B)** the micelles entered into tumor cells and concentrated on mitochondria by active targeting and released the drug to tumor cells (124).

In addition to attaching pH-sensitive acid-unstable bonds to the polymer backbone or side chains, there is another way to make the nano-drug carriers pH-responsive: introduce a chemical group into the nanomaterial that can lead to a change in surface charge properties (protonation or deprotonation) with the change in environmental pH. Such groups include amino, phosphoric acid, and carboxyl groups. These groups have different chemical structures and pKa values, can accept or provide protons, and undergo changes in physical or chemical properties related to pH, such as swelling or solubility, resulting in drug release (59, 60, 122). A nano-drug carrier has been designed, which has neutral or amphoteric ionic when it circulates in the human body, as this allows an improved circulation time for the nano-drug carrier in the body. When the nano-drug carrier reaches the tumor site, the surface charge of the nano-drug carrier is converted to positive, owing to the decreased pH in the tumor microenvironment, which promotes the uptake of tumor cells, thus greatly improving drug delivery efficiency (116, 123). For example, Fang et al. (124) coupled berberine (Ber) and vitamin B6 with oligomeric hyaluronic acid (OHA), creating self-assembled spherical NPs in an aqueous solution to prepare pH-responsive mitochondria-targeting NPs (B6-OHASS-Ber). In the acidic microenvironment of the tumor tissue, the pyridine group of B6 was protonated, and the surface of the carrier changed from negatively to positively charged, which promoted the internalization of NPs. After lysosome escape, it actively targeted mitochondria and released the drugs. The *in vivo* anticancer activity experiment results showed that B6-OHA-SS-Ber/Cur had an obvious inhibitory effect on tumor growth. As shown in **Figure 3**, the micelles were modified by B6 to give the NPs the ability to convert charges, Ber was induced to focus on mitochondria preferentially by drugs and carriers, and OHA was used to target CD44 receptors (125, 126).

In addition, there also exists a pH-responsive polymer that changes the hydrophilicity or solubility of nano-drug carriers after protonation with a decrease in pH, resulting in the change in carrier structure and drug release (127). For example, some polymers synthesized from acidic monomers, such as poly(methacrylic acid) (128, 129), poly(aspartic acid) (PAsp) (130), and sulfonamide-based polymers (131), change from hydrophilic to hydrophobic when the pH is decreased. Kang and Bae (132) studied oligomeric sulfonamides (OSASs), which could effectively release polymers or transport nucleic acids. Owing to the different pKa values of different sulfonamide groups, OSASs change from hydrophilic to hydrophobic with a decrease in the surrounding pH and show different proton buffering capacities. Kang and Bae (132) combined an OSAS with DNA and then complexed it with poly(L-lysine) (PLL) to form an OSA polymer. The experimental results showed that there was no significant difference in DNA uptake between the OSA polymer and PLL/DNA control group; however, the OSA polymer induced a more extensive distribution of DNA in cells and significantly enhanced the transfection rate of DNA.

### 3.3 Synergistic Action of pH Stimulus and Other Stimuli

We have previously discussed the two main mechanisms of action of pH-responsive polymers, namely, unstable chemical bonds with pH sensitivity and groups or polymers with charge transfer or water solubility changes. However, these pH-responsive polymers should remain stable when the physiological pH is 7.4, and it is challenging to ensure sufficient drug release rate or cell uptake rate with a decrease of 1 pH unit (60). At present, many preclinical studies are being conducted at two pH values (7.4 and 5.0). Nevertheless, if there is only a small difference between the targeted position of the nano-drug carriers and the physiological pH, the drug release time of polymers that solely depend on the pH-responsive mechanism, especially the nano-drug carriers that rely on covalent bond cleavage, will be very slow (60). Hence, compared to single stimulus-responsive nano-polymers, dual or multiple stimuli-responsive polymer NDDSs have higher physiological or biological system correlations, can better adapt to the interaction in the real biological system (133), and greatly improve the stability of drug delivery and the sensitivity of controlled release, giving excellent anti-tumor effects and broader application prospects (134).

Compared with normal tissues, tumor tissues show significantly different physiological characteristics, such as differences in pH, redox enzyme levels, ROS levels, owing to their uncontrolled growth and abnormal gene expression. We can select or combine the corresponding stimulus-response factors for tumor-targeted therapy according to specific changes in the tumor microenvironment. In addition, some polymers will change their morphological structure, physical, or chemical properties when exposed to external stimuli (temperature, light, magnetic field, and ultrasound), which can be combined with pH stimulation response for tumor treatment to improve the therapeutic effect (135–139).

**Figure 4:**
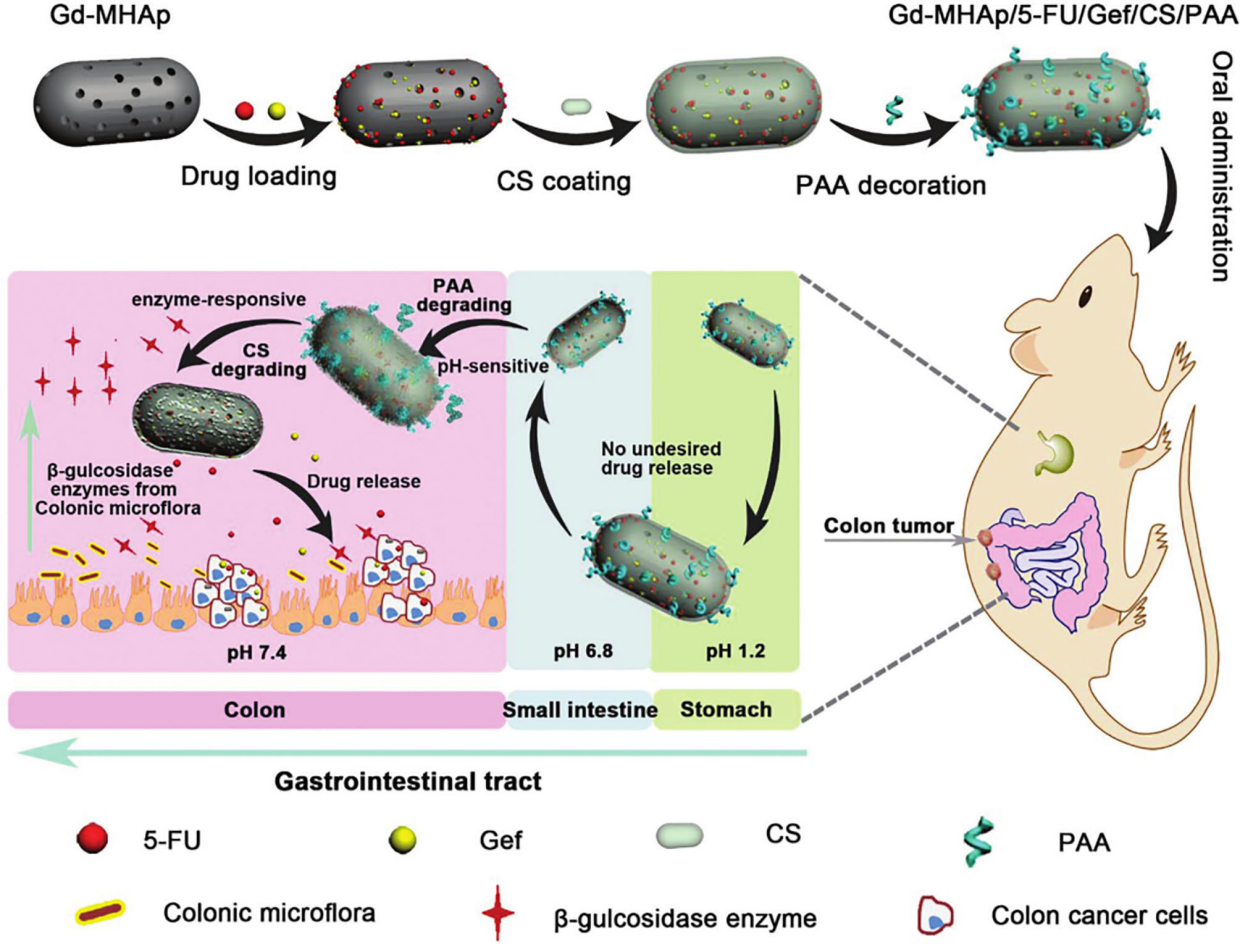
Schematic illustration of the preparation of Gd-MHAPNPs and the mechanism of drug release in different physiological environments in the GIT (140).

To reduce the problem of non-specific drug release in oral DDSs, Song et al. (140) studied the effect of pH response combined with enzyme response (double stimuli) on precisely targeted drug delivery. They anchored polyacrylic acid (PAA) and chitosan (CS) onto Gd3+-doped mesoporous hydroxyapatite NPs (Gd-MHAPNPs) to realize programmed drug release and magnetic resonance imaging at tumor sites. Among them, the grafted PAA acted as a pH-responsive switch to control the release of drugs into the colon. In addition, CS was functionally modified into enzyme-sensitive parts and could be degraded by β-glucosidase in the colon. Chemotherapeutic drugs (5-fluorouracil) and targeted therapy drugs (gefitinib) were encapsulated in Gd-MHAP NPs for synergistic therapy (**Figure 4**). The experimental results showed that CS and PAA protected drug-loaded NPs from various physiological conditions in the gastrointestinal tract, in order for the drug to reach the colon tumor site, prevent premature drug release, and control drug release *via* the dual stimulus-response mechanism (pH and enzyme), which increased the drug load at the colon tumor site and improved the therapeutic effect.

Owing to the high reducibility in the tumor environment, such as the increased glutathione (GSH) content, Kim et al. (141) designed pH- and GSH-responsive fluorescent polymer dots (L-PDs) containing PTX. The core part of the L-PD nano-hybrid system used disulfide bonds as the redox response site, which could not only maintain the stability of the internal structure but could also avoid the leakage of PTX during the cycle. The nano-hybrid system showed fluorescence quenching behavior, with less than 2% of the PTX being released under physiological pH conditions. However, in the tumor microenvironment, fluorescence was restored under the acidic pH, and the PTX release was about 100%, which significantly improved the effectiveness of killing tumor cells. In addition, the experimental results showed that there were differences in the release of PTX under different GSH concentrations, and the explosive release of PTX was detected under a high GSH concentration.

In addition, because of the high levels of reactive oxygen species (ROS) and low pH levels in the tumor microenvironment, Jäger et al. (142) designed functionalized “AND gate” multi-responsive (MR) block amphiphilic copolymers with dual stimulus-response to ROS and pH for cancer drug delivery therapy. The hydrophobic part of the polymer contained masked pH reaction side chains, which were exclusively exposed in response to the high ROS in the tumor microenvironment. These researchers synthesized two kinds of pH-responsive polymers from MR-ethyl and MR-isopropyl and then prepared DOX-loaded nano-drug carriers (multi-responsive nanoparticles, MRPs) and control-free DOX carriers using microfluidic self-assembly technology. The experimental results showed that compared to the control-free DOX carriers, the drug release rate of the MRP group was significantly faster under the influence of the dual ROS and pH response.

In addition to taking the different biochemical indexes of tumor tissue as stimulus-response factors, some polymer components undergo volume phase transition when the external environment, such as temperature, changes, thereby triggering the selective release of drugs. For example, Wang et al. (143) synthesized the temperature and pH double stimuli-responsive NPs consisting of deoxycholic acid and hydroxybutyl-decorated chitosan (DAHBCNPs) and designed a series of DAHBCs with different lower critical solution temperatures (LCSTs, all below 37°C) to load Cur for the oral treatment of colorectal cancer. Owing to the mutual offset of the size change between the polymer expansion after pH response and the polymer contraction after temperature response, the DAHBC NPs were relatively stable under simulated gastric conditions (pH 1.2, 37°C). However, in simulated intestines (pH 7.0–7.4, 37°C), DAHBC NPs promoted the continuous release of Cur into the intestinal tract on account of the single effect of polymer contraction after temperature response. Compared with free Cur, DAHBC NPs at 27°C (DAHBC27) were better absorbed by Caco-2 cells through transcellular and paracellular transport pathways, and the intestinal absorption efficiency of Cur was greatly improved by paracellular transport pathways. In addition, Chen et al. (144) Feng et al. (145), and Lin et al. (146) reported on multifunctional nano-drug carriers with dual pH and temperature-stimuli responses (photothermal therapy). For example, Chen et al. (144) prepared a multifunctional NDDS with pH and temperature response based on an ordered mesoporous silica-sandwiched black phosphorus nanosheet (BP@MS) with vertical pore coating. The special structure of the drug carrier not only enhanced the dispersity of the BP nanosheet and improved the drug loading efficiency (DOX), but also facilitated post-modification, such as the PEGlyation and conjugation of the ligand targeting TKD peptide. The results of *in vivo* and *in vitro* anti-tumor experiments showed that the DOX-loaded BP@MS system (BSPTD) manifested heat-stimulative, pH-responsive, and sustained release behaviors. Moreover, the BSPTD could also significantly inhibit the lung metastasis of tumors, owing to the secondary administration promoted by photothermal degradation.

In addition to dual stimuli, the combined application of multiple stimuli in tumor therapy has been widely studied. Don et al. (147) synthesized a poly(AA-b-NIPAAm) copolymer (PAA-b-PNIPAAm) *via* reversible addition–fragmentation chain transfer polymerization, consisting of PAA and poly(N-isopropylacrylamide) (PNIPAAm), which self-assembled to form nanogels with cationic protein (protamine). Chemical characterization showed that the nanogel exhibited a change in protamine conformation, PAA-b-PNIPAAm disaggregation, and protein enzymatic hydrolysis when stimulated by temperature, pH, and enzyme activity level changes. Changing the pH from 7.4 to 5.0-6.5 increased the mean particle size and converted the surface charge from negative to positive. After cold shock treatment, DOX was rapidly released in the cells. Furthermore, the nanogel could also carry a rose bengal photosensitizer and cause significant damage to cancer cells under irradiation.

### 3.4 Other Mechanisms

In addition to the two basic principles introduced above, another pH response mechanism has been gradually discovered and studied, the principle of which is that bicarbonate reacts with an acid to produce CO2 and H2O. Therefore, polymer nanomaterials containing bicarbonate structures may also release drugs specifically in an acidic environment, in what is known as a gas-generating pH-sensitive nano-system (59). Kim et al. (148) encapsulated poly(lactide-co-glycolide) (PLGA) NPs with sodium bicarbonate, which could be decomposed to produce CO2 under acidic conditions, and imidazoquinoline-based synthetic toll-like receptor 7/8 agonist (TLR7/8, also known as “522”). The addition of sodium bicarbonate not only significantly increased the loading dose of 522, but also decomposed and released CO2 through pH response after the NPs were internalized by tumor cells and phagocytized by lysosomes, resulting in the release of 522 into tumor cells. Finally, the experimental results also showed that the new pH-sensitive NPs had a better anti-tumor effect than traditional PLGA NPs.

## 4 Application of pH-Responsive Polymer Nanomaterials in Tumor Therapy

**Figure 5:**
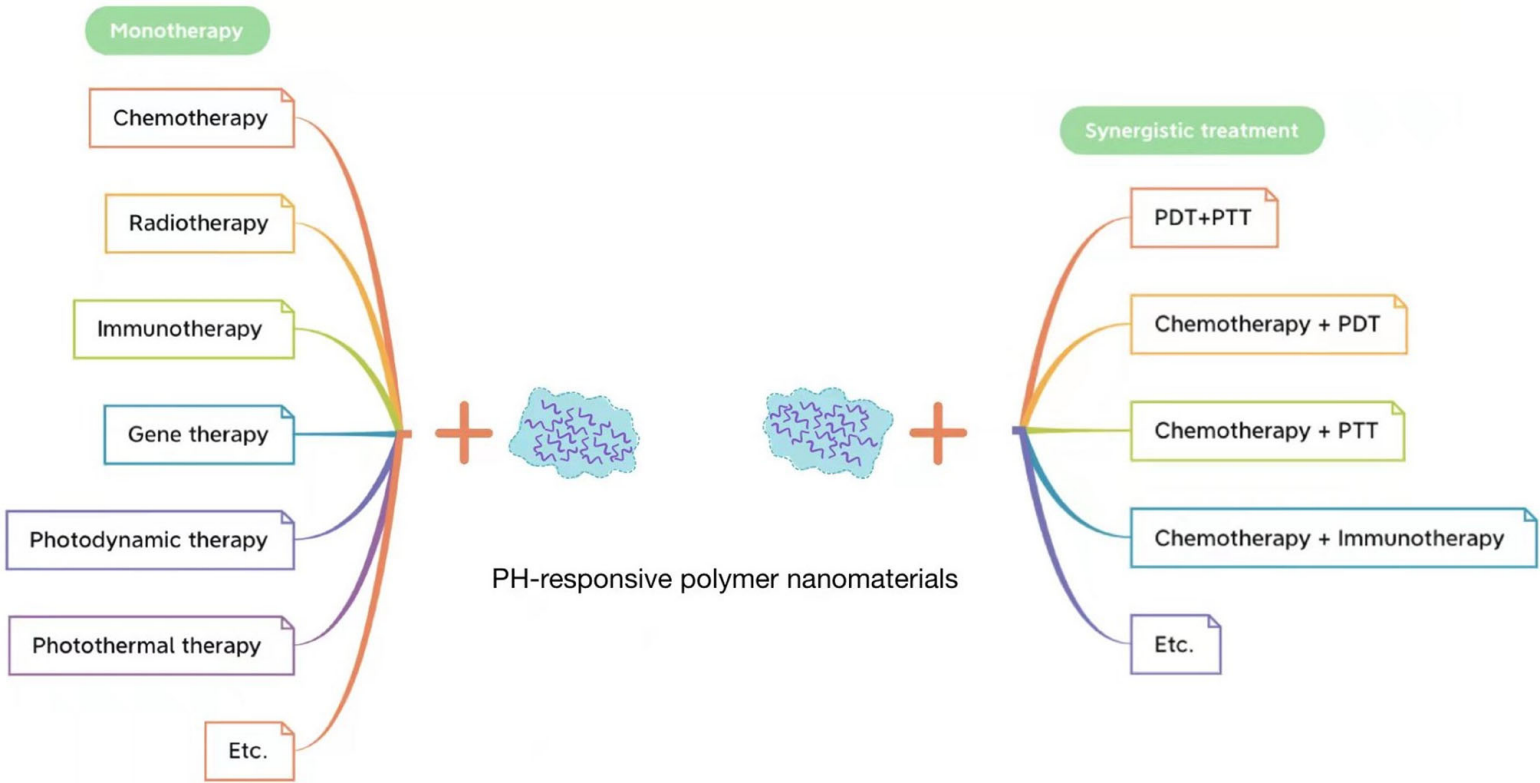
Application of pH-responsive polymer nanomaterials in tumor monotherapy (left) and application of pH-responsive polymer nanomaterials in tumor synergistic treatment (right).

At present, there are many effective treatment approaches for tumors, from surgical resection, chemotherapy, and radiotherapy to landmark molecular targeted therapy, immunotherapy, gene therapy, photodynamic therapy, photothermal therapy, and chemodynamic therapy, all of which have made great progress and development throughout the years. As is well known, the traditional administration of chemotherapy leads to the poor targeting of drugs to tumors, low drug utilization, and many other challenges. Therefore, scholars have proposed DDSs in which chemotherapeutic drugs are loaded and transported on carriers (149). To improve the efficiency of DDSs, it became a hot spot (150, 151) in tumor therapy research to design nano-drug carriers that can respond to the abnormal biochemical indicators in the tumor microenvironment (152) to release drugs directly into the tumor site. With the development of nanomaterials, nano-systems can enhance the permeability and retention of drugs in solid tumors (59) by introducing different stimulus-responsive structures into DDSs (153), among which pH-responsive polymers have been widely used in stimulus-responsive NDDSs (59, 100). The common applications of pH-responsive polymer nanomaterials in tumor therapy are summarized in **Figure 5**.

### 4.1 Application of pH-Responsive Polymer Nanomaterials in Tumor Monotherapy

#### 4.1.1 Application of pH-Responsive Polymer Nanomaterials in Combination with Chemotherapy

Single drug therapy will produce serious drug resistance in tumor treatment, but combinations of different drugs have been proven to be very effective in overcoming multidrug resistance (154, 155). Moreover, the combination of two or more drugs can generally reduce the drug dose and toxicity (156). Thus, constructing a multifunctional pH-responsive NDDS provides an effective strategy for chemotherapy. Wang et al. (157) synthesized multifunctional NPs (FTDCAG NPs), using a pH-responsive prodrug (PEG2K-NH-N-DOX), a GSH-responsive prodrug (PEG2K-S-S-CPT), folate-receptor targeting polymers (FA-PEG2K-L8, FA-PEG2K-TOS), and T1-enhanced magnetic resonance imaging contrast agents (Gd-DTPA-N16-16) used to encapsulate combrestatin A4 (CA4). These FTDCAG NPs contained three drugs, namely, CA4, DOX, and CPT. At pH 6.5, the drug release rate of CA4 increased by 63.4%. After release, CA4 destroyed angiogenesis in the tumor microenvironment and promoted the uptake of the remaining FTDCG NPs by tumor cells. After entering tumor cells, the FTDCG NPs disintegrated, owing to the increase in intracellular GSH concentration and the decrease in pH levels in the endosomes or lysosomes, releasing CPT and DOX. The results showed that FTDCAG NPs had a significant anti-tumor effect, and controlled drug release in time and space, which could maximize the effect and accuracy of cancer chemotherapy.

#### 4.1.2 Application of pH-Responsive Polymer Nanomaterials in Combination with Radiotherapy

Radiotherapy is the most effective method to treat malignant brain tumors, but it is a challenge to ensure minimum damage to the surrounding normal tissue, while providing the best therapeutic dose to the tumor tissue (158). Therefore, one of the basic treatments for glioblastoma is radiotherapy combined with radiosensitizers. Shirvalilou et al. (159) used 5-iodo-2-deoxyuridine (IUdR) as a radiosensitizer for glioblastoma and magnetic graphene oxide NPs coated with PLGA polymer as drug carriers for IUdR (MNPs). The results of *in vivo* and *in vitro* experiments showed that MNPs not only prolonged the circulation time *in vivo* but also targeted glioma through an external magnetic field after intravenous injection. Moreover, the experiment results illustrated a higher IUdR release rate at pH 5.6 than at pH 7.4, where 82% and 56% of the IUdR was released within 24<n,bsp/>h respectively. The drug release rate was significantly increased when the NPs reached the tumor site due to the degradation of PLGA polymers as a result of charge transfer under acidic pH conditions, revealing the controlled drug release abilities of the MNPs in the acidic environment of the tumor. Under high voltage X-ray irradiation, compared with the control group, IUdR/MNPs showed a stronger radiation sensitization effect and increased apoptosis induced by radiation, which improved the therapeutic effect.

#### 4.1.3 Application of pH-Responsive Polymer Nanomaterials in Combination with Immunotherapy

Compared with chemotherapy, immunotherapy has more advantages in terms of avoiding toxicity and side effects, inhibiting tumor metastasis, and preventing tumor recurrence; it is recognized as the next generation of tumor therapy (160, 161). The complement system plays an important role in promoting antigen-specific immune responses (162). Polymers carrying nucleophilic groups, such as hydroxyl or amino groups, can activate the complement system, resulting in the maturation of dendritic cells and induction of enhanced immune responses (163–165). Li et al. (166) prepared a multifunctional pH-responsive micelle vaccine platform (COOH-NPs) based on a carboxyl-modified deblock copolymer of poly(2-ethyl-2-oxazoline)–poly(D, L-lactide) (COOH-PEOz-PLA). After injection, the vaccine showed good targeting, and the tertiary amine groups in the PEOz backbone became protonated under acidic conditions, imparting the micelle payloads with endosomal escape *via* the **“**proton sponge**”** effect (167, 168). Compared with the control group, the nano-vaccine exhibited good laminin targeting, antigen presentation ability of cytotoxic T lymphocyte immune response, and complement activation to promote dendritic cell maturation, which significantly inhibited tumor growth and prolonged survival time.

#### 4.1.4 Application of pH-Responsive Polymer Nanomaterials in Combination with Gene Therapy

Traditional gene therapy generally adopts the method of enhancing host anticancer immunity, antagonizing antioncogenes, expressing suicide genes, or gene modification combined with high-dose chemotherapy. While achieving certain curative effects, it also has some disadvantages, such as low gene vector introduction efficiency and poor accuracy and controllability of foreign gene expression *in vivo*. With the development of genomics and genetic engineering research, recent new technologies, such as targeted gene-viro-therapy (169), RNAi technology (170), and microRNA (171), provide new bases or evidence for the accurate treatment of cancer. In gene therapy, hyperbranched PEI-25k and its derivatives have been widely developed and applied, owing to its high density of positive charges, which can bind closely to DNA and promote its escape from endosomes (172, 173). Chen et al. (174) synthesized a novel pH-responsive polymer shielding system (PAMT) *via* click reaction between poly(γ-allyl-L-glutamate) and thioglycolic acid or 2-(Boc-amino) ethanethiol. PAMT was electrostatically attached to the surface of the positively charged PEI/pDNA complex to form a ternary complex. When the pH in the external environment decreased from 7.4 to 6.8, the PAMT released gene therapy drugs through rapid charge conversion. The *in vivo* anti-tumor experiment showed that the transfection efficiency of the PAMT/PEI/shVEGF group was higher and the inhibitory effect on tumor growth was more obvious, compared to the control group.

#### 4.1.5 Application of pH-Responsive Polymer Nanomaterials in Combination with Photodynamic Therapy

In recent years, photodynamic therapy (PDT) has been widely used in tumor therapy. Earlier, Kelly et al. (175) successfully used hematoporphyrin derivatives as photosensitizers in the treatment of bladder cancer, creating a precedent for the application of PDT on tumors. The traditional DDSs relying on photosensitizers have limited application for the PDT of tumors, owing to their poor pharmacokinetics (176, 177) and long-term biological safety issues (176–178). For example, MoS2 DDSs, the biotoxicity of which is caused by quantum size effects and surface effects, may have adverse effects on the human reproductive system (178). Previous studies have shown that nanomaterial drug carriers have a higher uptake rate of tumor cells and fewer side effects (177), and the specific response of drugs to the tumor can be further enhanced by designing stimulus-responsive nano-drug carriers.

To overcome the shortcomings of the non-specific tumor therapy associated with PDT, Li et al. (179) and He et al. (180) applied nanomaterials with stimulus-response to PDT, which made the therapeutic drugs release specifically at the tumor site, reducing the non-specific phototoxicity of PDT and improving the anti-tumor effect. Ma et al. (181) combined chidamide as a histone deacetylase inhibitor (HADCi) with pH-responsive block polymer folate PEG-b-poly(aspartic acid) (PEG-b-PAsp)-grafted folate (FA-PEG-b-PAsp) to obtain block polymer folate PEG-b-poly(asparaginyl-chidamide) [FA-PEG-b-PAsp-chidamide (FPPC)]. Then the model photosensitizer pyropheophorbide a (Pha) was coated with FPPC and polymer micelles (Pha@FPPC) were formed in PBS. After the uptake of Pha@FPPC by tumor cells through FA-receptor-mediated endocytosis, PAsp changed from hydrophilic to hydrophobic in the lysosome due to the decrease in pH, which led to the directional release of Pha molecules. The results showed that Pha@FPPC had low dark cytotoxicity *in vitro* and a good therapeutic index, owing to its high dark cytotoxicity:photocytotoxicity ratio. Furthermore, compared with free Pha, Pha@FPPC not only significantly inhibited the growth of the implanted tumor and prolonged the survival time of melanoma-bearing mice, but also remarkably prevented the pulmonary metastasis of mice melanoma through folate-mediated and selective accumulation.

#### 4.1.6 Application of pH-responsive Polymer Nanomaterials in Combination with Photothermal Therapy

Similar to PDT, photothermal therapy (PTT) has become an effective method to treat cancer with high specificity, good anti-tumor effects, controlled drug release, and few side effects (154, 182, 183). As an NIR-absorbing organic photosensitizer, ICG is an amphiphilic tricarbocyanine dye that has been approved by the United States Food and Drug Administration for medical diagnosis and imaging. Although ICG has great potential in PTT applications, it also has many defects (184, 185). To overcome these shortcomings, pH-responsive polymer micelles or NPs have been used to design tumor-targeting ICG delivery methods. Ting et al. (186) co-assembled hybrid polymer NPs (PbCTPNs) comprising hydrophobic PLGA segments, ICG molecules, amphiphilic tocopherol PEG succinate (TPGS), and pH-responsive mPEG-benzoic imine-1-octadecylamine (mPEG-b-C18) segments. PbCTPNs maintained sufficient stability under physiological pH conditions, effectively inhibiting the self-aggregation and leakage of ICG. However, when the pH decreased to 5.5, the unstable imine bond in mPEG-b-C18 was hydrolyzed, which accelerated the release of ICG molecules in combination with near-infrared radiation, significantly killing MCF-7 cells and improving the anticancer efficacy of ICG-mediated PTT.

### 4.2 Application of pH-Responsive Polymer Nanomaterials in Tumor Synergistic Treatment

Previously, we discussed the application of pH-responsive polymers in tumor monotherapy, such as PDT, PPT, immunotherapy, and gene therapy; although they are still in the early clinical stages, they have shown good efficacy and clinical results. However, studies and clinical experiences have shown that it is difficult for monotherapy to eliminate tumors and control their metastasis; some cell subsets in unevenly distributed tumor tissues are resistant to monotherapy (187, 188). For example, in chemotherapy, long-term use of the same anticancer drug will induce tumor multidrug resistance, resulting in a decline in efficacy (189). Thanks to the rapid development of nanotechnology, we can combine two or more therapies and design multifunctional nano-drug carriers for the targeted delivery of multiple drugs, which can not only improve the curative effects, but can also reduce the side effects caused by high doses of a single therapy and make up for the defects and deficiencies of various other treatments (190, 191).

Photothermal therapeutic agents are also excellent photosensitizers, and the combination of PDT and PTT may have a synergistic effect on phototherapy. Bejjanki et al. (192) studied the efficacy of PTT combined with PDT in treating tumors. They combined ICG, Fe3O4, and palmitoyl ascorbic acid (PA) with pH-responsive PEG-b-PAEMA-PDMA polymers to form nano-drug carriers. The carriers remained stable under physiological pH and the cycle time was prolonged, while drug loading was released *via* charge conversion and tumor cell uptake was promoted in the acidic tumor environment. The results of *in vivo* and *in vitro* tumor cell experiments showed that the nano-drug carrier showed a synergistic PDT/PTT effect, had a high apoptosis rate, and significantly inhibited tumor growth.

Compared with photodynamic therapy, chemodynamic therapy is a new treatment strategy (193). It can kill tumor cells by converting overexpressed hydrogen peroxide into highly toxic hydroxyl free radicals through Fenton and Fenton-like reactions. It has a high tumor selectivity (194, 195). Wang et al. (196) reported a pH and ROS dual-response nano-drug (PtkDOX-NM), which accumulates in the tumor site through the enhanced permeability and retention effect. In the acidic endosomal environment, the poly(2-[diisopropylamino] ethylmethacrylate) segment of the drug changes from hydrophobic to hydrophilic, resulting in β-lapachone (Lap) dissociation and rapid release. The Lap promoted the production of hydrogen peroxide, and a Fenton reaction occurred in the presence of iron ions, which significantly increased the ROS content in the tumor environment, thus promoting the effective cascade release of antineoplastic drugs in response to ROS and enhancing the curative effect through the combination of chemodynamic therapy and chemotherapy.

**Figure 6:**
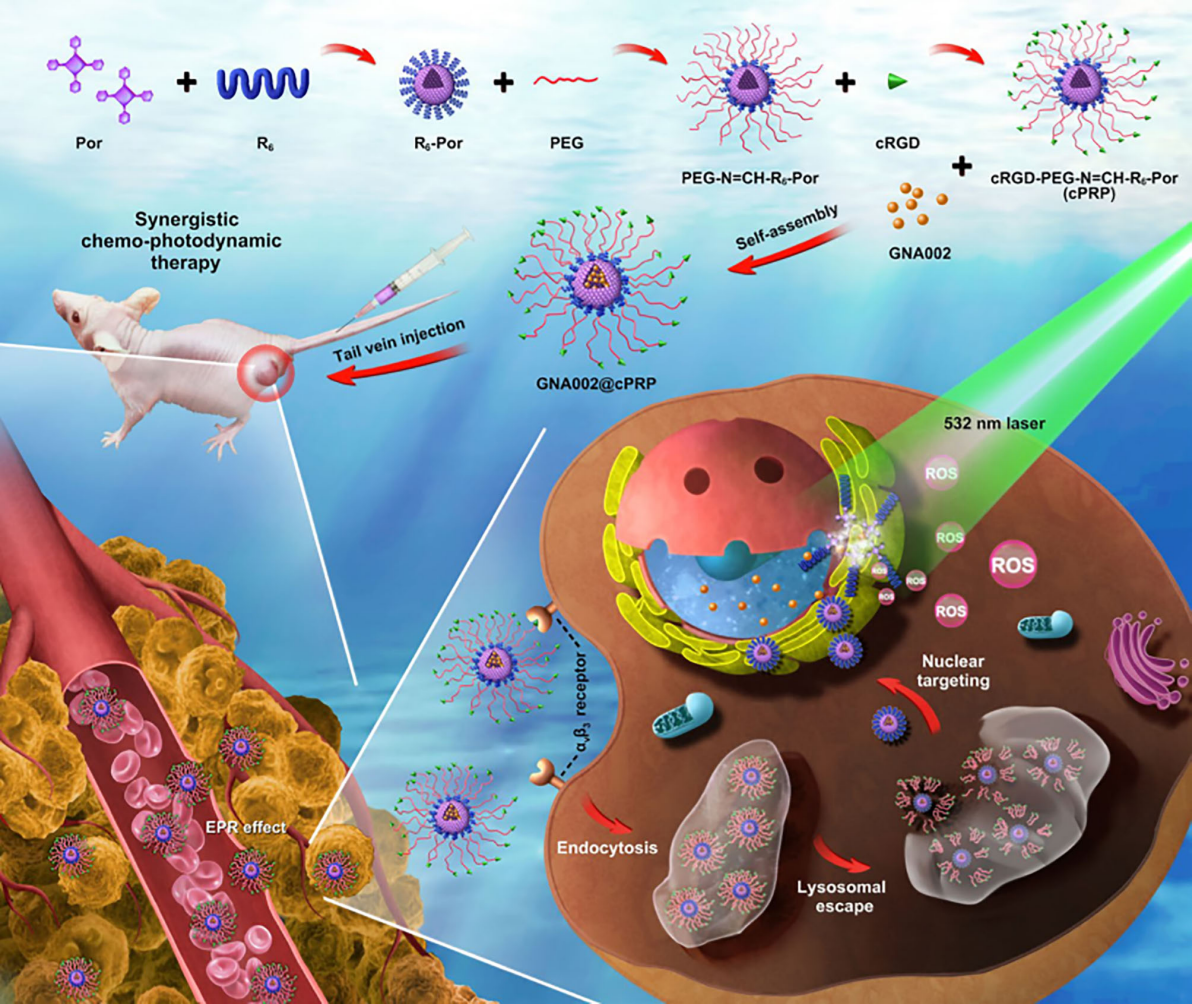
Schematic illustration of a pH cascade-responsive nucleus-targeted nanoplatform for synergistic chemo-photodynamic therapy (197).

To verify the synergistic effect between chemotherapy and PDT therapy, Li et al. (197) combined anti-tumor drug GNA002-mediated chemotherapy with PDT therapy through an NDDS, which improved the efficacy of anticancer therapy. They used 5-(4-carboxyphenyl)-10,15,20-triphenylporphyrin as the photosensitizer and GNA002 as the hydrophobic core and cyclic RGD peptide (cRGD)-modified PEG (cRGD-PEG) connected to cell-penetrating peptide hexa-arginine (R6) through an unstable hydrazone bond as a hydrophilic shell to synthesize a nucleus-targeted pH cascade-responsive micelle nano-platform (**Figure 6**). The NPs were accumulated at the tumor site, and then actively internalized into tumor cells through the cRGD ligands. In cells, when the NPs were swallowed by lysosomes, the hydrazone bond was hydrolyzed and the shell fell off due to the change in pH. Then nucleus-targeted drug delivery mediated by R6 was treated with chemotherapy and PDT under laser irradiation.

In addition, Tang et al. (198) studied whether there was a synergistic effect between chemotherapy and PTT. They designed a biosensor with a MOF coated with multifunctional polydopamine (PDA) for the detection of miRNA-122 with Zn2+-triggered aggregation-induced enhancement (AIE) and synergistic chemotherapy/PTT. They used ZIF-8 NPs as DOX carriers and modified them with polydopamine to improve the pH response and biocompatibility of the nano-drug carriers. When the nano-drug carrier reached the tumor site, the acidic environment led to a change in the carrier structure and accelerated the release of Zn2+ and DOX. The experimental results not only showed that the nano-drug carrier accurately detected miRNA-122, but also verified the synergistic effect of chemotherapy and PTT on tumor therapy.

To solve the multidrug resistance of chemotherapeutic drugs, the combination of chemotherapy and other therapeutic methods for treating tumors has been widely studied and explored. For instance, Wang et al. (199) studied whether there was a synergy between chemotherapy and tumor immunotherapy. They employed a unique cationic switchable lipid as a pH-responsive drug carrier, which encapsulated the anti-tumor drug sorafenib and was loaded with anti-miRNA27a-loaded anti-GPC3 antibody to form targeted lipid NPs (G-S27LN), enabling targeted release the sorafenib and antibodies in the acidic environment of the tumor. Compared to the control group, the survival rate of tumor cells decreased significantly, and the proportion of apoptotic cells was remarkably higher than that in the control group, showing a synergistic therapeutic effect.

## 5 Conclusion and Outlook

pH-responsive polymer nanomaterials can stably carry anti-tumor drugs through the body and release them into tumors through acid-unstable bond fracture or charge conversion for certain targeted therapeutic effects. Compared with conventional tumor chemotherapy, treatment with pH-responsive polymer nanomaterials exhibits improved therapeutic efficiency with minimal invasiveness to the host organism, which highlights its great potential in clinical trials or preclinical studies. Despite the notable progress made in the field of pH-responsive polymer nanomaterials, there are many critical issues that remain to be solved for better clinical application. Therefore, the potential focuses and challenges of pH-responsive polymer nanomaterials have been discussed below.

(1) Few pH-responsive polymer nanomaterials are currently available for clinical use, owing to the enhanced permeability and retention effect, as well as other delivery difficulties (200–204). These polymer nanomaterials are delivered through five stages in tumor therapy: being injected intravenously into the bloodstream, accumulating specifically around the tumor tissue, entering into the solid tumor, cellular internalization, and “CAPIR”, i.e., “circulation, accumulation, penetration, internalization, drug release.” In the current research stage, it is impossible to consider these five stages, and it is still impossible to achieve the complete treatment of tumors (205–207). This must be the main focus of future research.

(2) Further research is needed to achieve the precise targeting of pH-responsive polymer nanomaterials, to realize an accurate combination of pH-response stimuli and other response stimuli, to optimize pH-responsive stimulation as a tumor therapy, as well as to realize the multifunctional platform function of pH-response stimulation for tumor treatment.

(3) There are some crucial issues associated with pH-responsive polymer nanomaterials, especially those containing synthetic drugs or their derivatives, including manufacturing technique problems, safe application problems, tumor treatment evaluation criteria accuracy problems, and institutional problems, which should be resolved as soon as possible to favor the successful clinical application of treatments after fundamental research is completed. More attention should be paid to establishing the entire standard treatment process, systematic evaluation, and long-term observation.

(4) Finally, the acute short-term toxicity and chronic long-term toxicity of pH-responsive polymer nanomaterials must be considered for the biological safety of clinical application in the future. These nanomaterials should be deeply studied to meet safety standards. At present, some suggested polymer nanomaterials are poisonous to the cells, causing side effects and other disease complications. For example, as mentioned earlier, the polymer nanomaterials containing hydrazone bonds may produce cationic polymer residues after hydrolysis *in vivo*, which are toxic to normal human tissues to some extent. Accordingly, new pH-responsive polymer nanomaterials, which are biodegradable and harmless to the body, should be developed to avoid such side effects. Sustainable materials, such as CO2 copolymers, should be further studied, with focus on ensuring that these materials undergo degradation through the Krebs cycle, metabolize into CO2 and water, and be excreted in a harmless manner *via* exhalation and through the action of kidneys.

In conclusion, pH-responsive polymer nanomaterials possess great potential for tumor therapy, and we believe that their beneficial effects will be recognized on a greater scale with the development of new technology and ongoing research.

## Author Contributions

FG contributed to the research retrieval and outline drafting. SC, XS and YT contributed to the research retrieval and drafting and critically revised the manuscript. SC critically revised the manuscript. Every author gave final approval and agreed to be accountable for all aspects of the work. All authors have read and agreed to the published version of the manuscript.

## Funding

Financial support from the Projects directly under Jilin Provincial Department of Finance, China (JCSZ2019378-23), and the Scientific Research Project of Jilin Provincial Department of Education, China (JJKH20211215KJ)

## Acknowledgments

Authors are thankful to their respective departments/institutes/universities for providing space and other necessary facilities, which helped to draft this manuscript.

## Conflict of Interest

The authors declare that the research was conducted in the absence of any commercial or financial relationships that could be construed as a potential conflict of interest.

## Publisher’s Note

All claims expressed in this article are solely those of the authors and do not necessarily represent those of their affiliated organizations, or those of the publisher, the editors and the reviewers. Any product that may be evaluated in this article, or claim that may be made by its manufacturer, is not guaranteed or endorsed by the publisher.
